# Differential Behaviours and Preferential Bindings of Influenza Nucleoproteins on Importins-α

**DOI:** 10.3390/v12080834

**Published:** 2020-07-30

**Authors:** Amélie Donchet, Emilie Vassal-Stermann, Francine C. A. Gérard, Rob W. H. Ruigrok, Thibaut Crépin

**Affiliations:** Centre National de la Recherche Scientifique (CNRS), Institut de Biologie Structurale (IBS), University Grenoble Alpes, Commissariat à l’Energie Atomique et aux Energies Alternatives (CEA), 38044 Grenoble, France; amelie.donchet@ibs.fr (A.D.); emilie.stermann@ibs.fr (E.V.-S.); francine.baraggia@grenoble-inp.fr (F.C.A.G.); rob.ruigrok@ibs.fr (R.W.H.R.)

**Keywords:** influenza nucleoprotein, nuclear transport, importin-α, nuclear localization signal, influenza-host interaction, surface plasmon resonance, fluorescence anisotropy

## Abstract

Influenza viruses are negative single-stranded RNA viruses with nuclear transcription and replication. They enter the nucleus by using the cellular importin-α/-β nuclear import machinery. Influenza nucleoproteins from influenza A, B, C and D viruses possess a nuclear localization signal (NLS) localized on an intrinsically disordered extremity (NP_TAIL_). In this paper, using size exclusion chromatography (SEC), SEC-multi-angle laser light scattering (SEC-MALLS) analysis, surface plasmon resonance (SPR) and fluorescence anisotropy, we provide the first comparative study designed to dissect the interaction between the four NP_TAILs_ and four importins-α identified as partners. All interactions between NP_TAILs_ and importins-α have high association and dissociation rates and present a distinct and specific behaviour. D/NP_TAIL_ interacts strongly with all importins-α while B/NP_TAIL_ shows weak affinity for importins-α. A/NP_TAIL_ and C/NP_TAIL_ present preferential importin-α partners. Mutations in B/NP_TAIL_ and D/NP_TAIL_ show a loss of importin-α binding, confirming key NLS residues. Taken together, our results provide essential highlights of this complex translocation mechanism.

## 1. Introduction

Influenza viruses are part of the *Orthomyxoviridae* family and form the genus *Influenzavirus*. Up to now, the International Committee officially recognizes four types (respectively types A, B, C and D) in its Taxonomy of Viruses (ICTV), but because of actual large-scale meta-transcriptomic approaches new specimens are regularly discovered, making this classification a constantly evolving entity. While influenza viruses were thought to be uniquely warm-blooded organism pathogens, a study has recently uncovered their existence in fish and amphibians in China [1]. A first criterion of distinction between the actual four types of *Influenzavirus* is based on their tropism. Influenza type A viruses (IAV) are viruses from water fowl but some of the viruses can also infect a range of hosts from chickens to swine and humans; type B (IBV) almost exclusively infect humans; type C (ICV) can infect both human and swine whereas type D (IDV) has a broad host tropism but limited to mammals. Influenza viruses are negative-sense single-stranded RNA viruses with segmented genomes. The RNA segments are packaged in ribonucleoproteins (RNPs), a complex architecture made by a single RNA molecule encapsidated by multiple copies of the nucleoprotein (NP) and the heterotrimeric RNA-dependent RNA polymerase (RdRp) bound to the strictly conserved 3′ and 5′ end [2,3]. The number of RNPs varies depending on the type: IAV and IBV genomes consist of eight RNA segments, i.e., there are eight different RNPs, but only seven for ICV and IDV viruses. This is related to the inherent organisation of the viral spikes, respectively the haemagglutinin (HA) plus the neuraminidase (NA) for A and B or a single hemagglutinin-esterase-fusion (HEF) polypeptide with both concatenated activities for C and D.

Unlike most RNA viruses, influenza viruses transcribe and replicate their genome in the nucleus of infected cells. In general, the access to the nuclear compartment is through the highly regulated nuclear pore complex (NPC) [4,5,6,7]. Several pathways are involved in cytoplasmic-nuclear trafficking. Small molecules (< 30–60 kDa) are able to diffuse passively through the NPC but bigger molecules often require an active mechanism to be imported [8,9,10]. The main active transport is based on the importins-α/β (coupled to the small GTPase Ran) pathway. Importins-α and -β can both interact with the cargos but only the last ones mediate directly the translocation, and importin-α:cargo complexes must first interact with importin-β [11,12]. Cargos are recognized by importins-α/β through nuclear localization signals (NLSs), usually made of basic or hydrophobic motifs [13,14]. Additionally to the interaction with the NLS motifs, recent karyopherins-β/cargo-protein structures have demonstrated that these complexes are dependent on multiple interactions, mediated through intricate 3D interfaces which involve residues well beyond the NLS peptide stretch [15,16,17].

**Figure 1:**
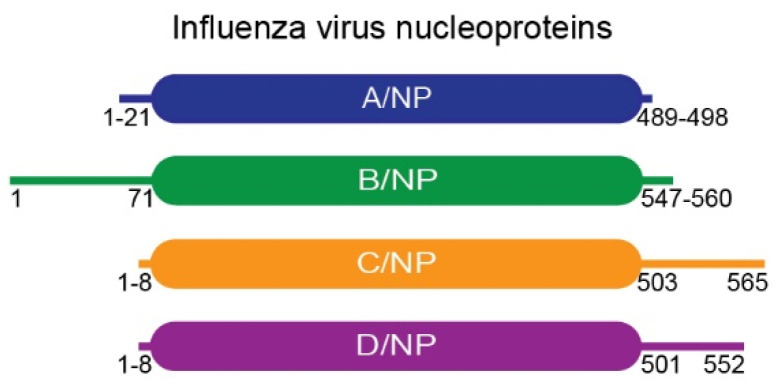
*Influenzavirus* nucleoproteins. The schematic representations of *Influenzavirus* nucleoproteins are based on this structural analysis [37]. The schema respects the size of the proteins. The flexible tails with the NLSs are represented with simple lines whereas the cores are represented with filled boxes.

The human genome encodes for seven importins-α and at least twenty importins-β [9,18,19], meaning that the importins-α/β pathway is a difficult problem. Nuclear import of influenza virus proteins has been of a major interest for a long time [20,21]. Considering the neo-synthetized components of the replication machinery, two subunits (PA and PB1) of the heterotrimeric RdRp are imported as a sub-complex by the importin-β RanBP5 [17,22,23,24] whereas the third subunit (PB2) and NP interact with importins-α [25,26,27,28]. Because of its role in the RNPs formation, NP is critical for influenza replication but also shown to be the major contributor to the nuclear transport of the RNPs [29]. The NLS of NPs are located in intrinsically disordered tails of the proteins, either in N_terminal_ for A/ and B/NP or in C_terminal_ for C/ and D/NP (Figure 1). A minor putative NLS has been located in a loop of the core of A/NP, between residues 198 to 216, but its role remains unclear [30,31]. A/NP is known to interact with several importin-α isoforms, including the universal -α1, -α3, -α5 and possibly -α7 [32,33,34,35,36]. In recent years, our group has worked on the mechanisms of the interaction between two different NPs and the human nuclear trafficking machinery [37,38], in particular the tails containing the NLS of influenza B (B/NP_TAIL_) and D (D/NP_TAIL_) NPs. We showed that the NLS of B/NP_TAIL_ is highly extended from residues 30 to 71, around a single basic Lysine-Arginine (44-KR-45) patch, with a low affinity of 844 nM for the human importin-α7. In the case of the C_terminal_ D/NP_TAIL_, the NLS was composed of two basic KR patches, respectively 514-KR-515 and 532-KR-533. Both contribute to the nuclear trafficking and we estimated that the affinity of D/NP_TAIL_ for the human importin-α7 was at 100 nM by surface plasmon resonance (SPR). The literature also contains several studies detailing the relation between A/NP and C/NP and importins-α [21,29,31,33,39,40,41,42,43] (Table 1). However, recent work highlighted that each importin-α is unique, with its own features and preferential partners [28,35,44,45].

Because we were confronted with a wide range of values (from 26 nM to 72 µM) obtained using different techniques and a plethora of different partner constructs, we wanted to establish a systematic comparative study of the in vitro interaction between the flexible extremities (NP_TAILs_) of all types of influenza NPs and the different human importin-α isoforms described in the literature. Our strategy was design, with a specific focus upon the short NLS sequences, and built with the objective of avoiding the inherent oligomerization of NPs, while guaranteeing the architecture of the tails in a unique globular environment. Using size exclusion chromatography experiments, we show that all NP_TAILs_ interact with importins-α in solution and then used surface plasmon resonance measurements to determine the affinity and binding kinetics of these interactions. In the current paper, we show that D/NP_TAIL_ interacts with all importins-α isoforms with strong affinity. Both A/NP_TAIL_ and C/NP_TAIL_ interact with varying affinities with the corresponding importins-α. On the other hand, B/NP_TAIL_ shows a completely different behaviour, with even faster association and dissociation rates and a low affinity with all the human importins-α we measured.

## 2. Materials and Methods

### 2.1. Molecular Biology and Constructs

**Figure 2:**
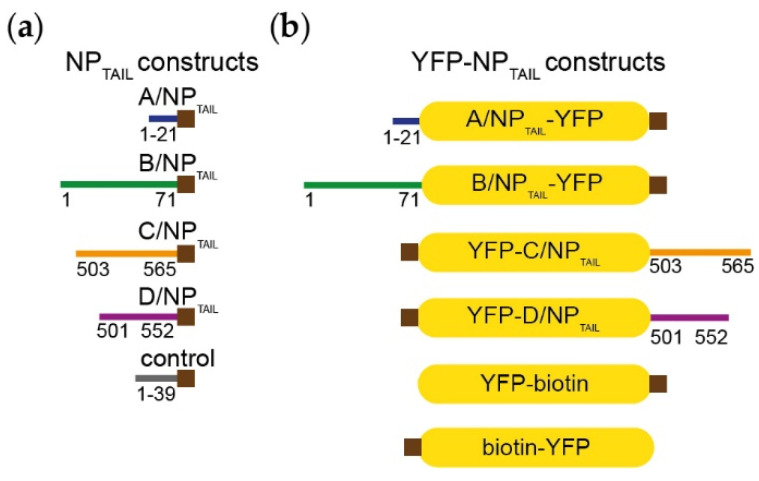
Constructs. (**a**) *Influenzavirus* nucleoproteins tails were expressed and purified independently fused with a biotinylation sequence (brown squares) in their C_terminal_ end. The details of the sequences are provided in Table S1. (**b**) The corresponding tails were fused to the yellow fluorescent protein (YFP) in order to integrate then in the context of a globular protein. The yellow filled boxes represent the YFP and the brown squares indicate the position of the biotinylation sequence.

Human importin-α1 (residues 69 to 529), -α3 (residues 64 to 521) and -α5 (residues 66 to 538) DNA coding sequences were bought at GENEART GmbH (Thermo Fischer Scientific, Regensburg, Germany) and subcloned into pETM11 bacterial expression vector (EMBL) without their importin-β (IBB) domains. Human importin-α7 (residues 58 to 536) cloning was described in [26]. NLS-bearing NP_TAILs_ from strains A/WSN/1933 (residues 1 to 21), B/Memphis/13/03 (residues 1 to 71), C/Ann-Arbor/1/50 (residues 508 to 565) and D/bovine/France/2986/2012 (residues 505 to 552) were ordered from GeneART, fused to a biotinylation sequence in C_terminal_ (Figure 2, and details of the sequences are provided in Table S1). The NP_TAILs_ were fused to the yellow fluorescent protein (YFP) either in C_terminal_ (C/NP_TAIL_ and D/NP_TAIL_) or in N_terminal_ (A/NP_TAIL_, B/NP_TAIL_) according to their native localization; at the other YFP extremity, the biotinylation sequence was added (Figure 2). NP_TAILs_-YFP constructs were subcloned into pETM11 bacterial expression vector. All constructs were expressed as N-terminally His-tagged proteins. Sequencing was performed by Eurofins Genomics (Köln, Germany). The control peptide was a synthetic peptide (GENPEP, St-Jean-de-Védas, France) corresponding to a GLA domain-derived polypeptide of the human coagulation factor X [46].

### 2.2. Expression and Purification

*Escherichia coli* BL21 RIL (DE3) cells (Life Technologies, Thermo Fischer Scientific, Courtaboeuf, France) were used for protein expression. Cultures were supplemented with biotin (12.5 μg·mL^−1^, Sigma, Saint-Quentin-Fallavier, France) to biotinylate the proteins *in cellulo*, induced for 12 h by adding 0.3 mM isopropyl-β-D-thiogalactopyranoside (IPTG; Euromedex, Souffelweyersheim, France) at 18 °C and collected by centrifugation. Pellets were resuspended in 50 mM Tris-HCl pH 7.5, 300 mM NaCl, 2 mM β-mercaptoethanol (β-ME; Roth, Lagny-sur-Marne, France) and cOmplete™, ethylenediaminetetraacetic acid (EDTA)-free protease inhibitor cocktail (Roche, Meylan, France) before sonication. All purifications included a nickel affinity chromatography (resin Ni-NTA; Qiagen, Les Ulis, France), a Tobacco Etch Virus (TEV) protease cleavage, a second nickel affinity chromatography and a size-exclusion chromatography (SEC). SEC was performed on a NGC system (Bio-Rad, Marnes-La-Coquette, France) with a S75 10/300 GL column (GE-Healthcare, Dutscher, Brumath, France). The final buffer composition for all proteins was 10 mM Hepes pH 7.5, 150 mM NaCl. NP_TAILs_-YFP protein concentrations were determined by measuring the absorbance at 280 nm and using the theoretical extinction coefficients (ε) 28,942 M^−1^·cm^−1^ for A/NP_TAIL_-YFP and C/NP_TAIL_-YFP, of 27,452 M^−1^·cm^−1^ for B/NP_TAIL_-YFP, D/NP_TAIL_-YFP and YFP control. ε at 280 nm used for ΔIBB importins-α protein concentrations were 50,607 M^−1^·cm^−1^ for importin-α1, 42,190 M^−1^·cm^−1^ for importin-α3, 52,285 M^−1^·cm^−1^ for importin-α5 and 46,785 M^−1^·cm^−1^ for importin-α7. The concentrations were then obtained using the Beer–Lambert law.

### 2.3. Interaction Assays by Size Exclusion Chromatography

All size exclusion chromatography (SEC) experiments were performed in the same buffer (10 mM Hepes pH 7.4, 150 mM NaCl) using a Superdex^TM^ 200 increase 10/300GL column (GE-Healthcare) for YFP-NP_TAIL_ fusion proteins and a Superdex^TM^ 75 10/300GL column (GE-Healthcare) for all the other NP_TAILs_. Samples were diluted to be used at 30 μM for YFP-NP_TAIL_ fusion proteins, 60 μM for NP_TAILs_ and 25 μM importin-α7 in 300 μL. They were incubated for one hour at room temperature before injection on a NGC system (Bio-Rad).

### 2.4. SEC-MALLS-RI Analysis

Size exclusion chromatography (SEC) followed by multi-angle laser light scattering (MALLS) and refractometry (RI) analysis allow the determination of the molecular mass of a protein or a complex in solution that is independent of its dimensions and shape [47]. SEC was performed with a column (Superdex^TM^ 200 increase 10/300 GL or Superdex 75 10/300 GL) equilibrated with 10 mM Hepes pH 7.5, 150 mM NaCl. Analytical runs were performed at 20 °C with a flow rate of 0.5 mL·min^−1^. Multi-angle laser light scattering (MALLS) detection was performed with a DAWN-HELEOS II detector (Wyatt Technology, Toulouse, France) using a laser emitting at 690 nm and protein concentration was measured on-line with the use of differential refractive-index measurements, with an Optilab T-rEX detector (Wyatt Technology) and a refractive-index increment, dn/dc of 0.185 mL·g^−1^. Data were analyzed and weight-averaged molar masses were calculated using the ASTRA software (Wyatt Technology Corp., Santa Barbara, CA, USA).

### 2.5. Surface Plasmon Resonance

Surface plasmon resonance (SPR) experiments were carried out at 25 °C on a Biacore T200 (GE Healthcare Life Sciences Europe GmbH, Velizy-Villacoublay, France). Avidin (50 µg·mL^−1^ in 50 mM acetate buffer pH 4.5; Sigma) was first immobilized on a Serie S CM5 sensor chip (GE-Healthcare) surface through amine coupling chemistry according to the manufacturer instructions, until a coupling level of about 17,000 resonance units (RUs) was reached. The biotinylated NP_TAIL_-YFP (1–10 μg·mL^−1^) or biotinylated YFP control (1–10 μg·mL^−1^) were then captured in surfactant-supplemented HBS-N running buffer (10 mM Hepes pH 7.4, 150 mM NaCl, 0.05% Tween 20; GE Healthcare) at a flow rate of 30 μL·min^−1^ until a coupling level of 100–200 RU was obtained. A flow-cell with immobilized avidin only was used as negative control while the flow-cell with the avidin: biotinylated NP_TAIL_–YFP complex was used as active flow-cell. For kinetic measurements, importins-α were serial-diluted in surfactant-supplemented HBS-N running buffer. Each concentration was injected in triplicate on both the reference and active flow-cells. Analyte injection and following buffer injection times were set between 55–180 s and 55–200 s respectively, depending on the examined partners, at a flow-rate of 30 μL·min^−1^. Regeneration was required and achieved by pulse injection of 5 M NaCl. All sensorgrams were reference-subtracted. Data were analyzed with Steady State Analysis using the Biacore T200 Evaluation software (GE Healthcare) under a Langmuir 1:1 binding model, assuming that the binding is equivalent and independent for all binding site. Chi^2^ values for the fitting were kept below 1 for most of the analysis with a maximum of 2.5, and T-value means for the dissociation constant above 10.

### 2.6. Fluorescence Anisotropy

Fluorescence anisotropy experiments were performed on a Clariostar microplate reader (BMG Labtech, Champigny-sur-Marne, France) set and dedicated to fluorescence anisotropy measurements, with excitation and emission wavelengths fixed at 482 and 530 nm, respectively. Importin-α1 was serially diluted in buffer (10 mM Hepes pH 7.4, 150 mM NaCl) and mixed in dilution series with the YFP-fused NP_tails_. YFP construct final concentration was set at 25 nM. Measurements were done in 384-well plates at room temperature. Blank substraction was done using the condition with YFP-fused NP_tails_ alone. Data were normalized using the equation:

(1) $$B=\frac{\left(Aexp-Amin\right)}{\left(Amax-Amin\right)}$$

where *B* corresponds to the YFP-NP_TAIL_ bound fraction (value between 0 and 1), *Aexp* is the anisotropy value observed for one given importin-α1 concentration, and *Amin* and *Amax* stand for the fluorescence anisotropy values of the free YFP-NP_TAIL_ and bound YFP-NP_TAIL_, respectively. Titrations were then fitted to the GraphPad Prism model “Single binding site with Hill slope (h)”.

## 3. Results

### 3.1. Biochemical and Biophysical Analysis of the Interaction between NP_TAILS_ and Importin-α7

In all experiments with NP and its partners, measurements are difficult because all NPs tend to form oligomers depending on the buffer concentrations [41,48], and therefore, we used only the disordered NP_TAILs_. We designed our study with a specific focus upon these short sequences that do not address the effect of domains beyond the NP_TAILS_. In order to decipher the specificities of the different NP_TAILs_ for importins-α, we first use the corresponding peptides, in size exclusion chromatography (SEC) experiments combined with surface plasmon resonance (SPR) measurements to determine the affinity and binding kinetics. The first method was used to confirm the ability of the tails to interact with its partner in solution, whereas the second method was used for the determination of the kinetic parameters of the interactions. In SPR, one partner (called the ligand) is immobilized onto a biosensor surface. The interacting partner (called the analyte) diluted in a buffer is then continuously flowed across the biosensor surface, where it binds to the ligand. The binding is measured as a change in resonance units (RUs) on the biosensor surface. Measuring the increase in binding over time for a given analyte set of concentrations gives the association rate (*k_on_*) up to the point where the system is at equilibrium (i.e., as many association events are observed as dissociation events). Ceasing flow and changing to buffer alone then allows the analyte to wash off the ligand. Measuring the decrease in bound partner over time gives the dissociation rate (*k_off_*). The equilibrium dissociation constant (*K_d_*) is calculated from the kinetic association and dissociation rates (*k_off_*/*k_on_*). When both association and dissociation rates are too fast, it is possible to use the equilibrium part of the curves to extract the dissociation constant, without relying on the rates. For the SPR experiments, we chose to immobilize the tails on the biosensor surface by using the importins-α as the analytes. The immobilization of the tails was done by fusing a biotinylation sequence in C_terminal_ (Figure 2a), with a capture strategy based on a biotin: avidin interaction. The corresponding constructs were recombinantly produced in bacteria (with 12.5 μg·mL^−1^ biotin in the growth media) and purified. We first tested their ability to interact with importin-α7 by SEC experiments. They all formed a stable complex in solution with importin-α7 (Figure S1). However, for SPR measurements, the kinetic parameters could not all be established because of technical issues. In particular, for B/NP_TAIL_, the inability to stabilize the SPR signal because of a putative mass transfer phenomenon made it impossible to exploit the raw data. In consequence, we adapted our strategy.

### 3.2. Biochemical and Biophysical Analysis of the Interaction between NP_TAILS_ Fused to Yellow Fluorescent Protein (YFP) and Importin-α7

**Figure 3:**
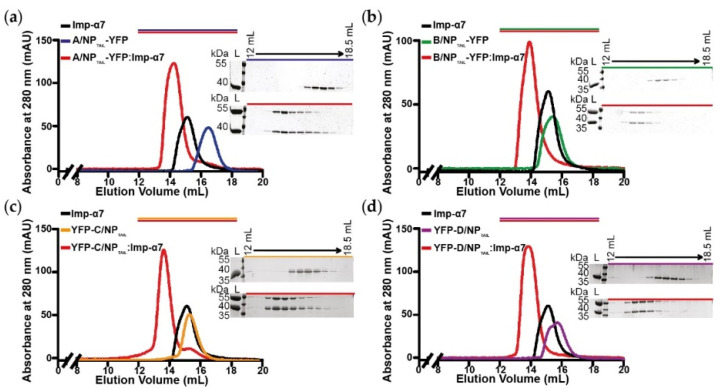
Analysis of the complexes between YFP-NP_TAILs_ and human importin-α7 in solution. Each YFP-NP_TAIL_ fusion protein (30 µM) was injected on a Superdex^TM^ 200 increase 10/300GL column, alone or in the presence of importin-α7 (25 µM). The figure shows the superimposition of the SEC profiles obtained for (**a**) A/NP_TAIL_-YFP, (**b**) B/NP_TAIL_-YFP, (**c**) YFP-C/NP_TAIL_ and (**d**) YFP-D/NP_TAIL_. The colors correspond to the code used on Figure 2b with all the complexes in red and the importin-α7 alone in black. For each panel, the 12% SDS-PAGE corresponding to the elution of the YFP-NP_TAIL_ fusion protein alone (top) and its complex with importin-α7 (bottom) are shown. The control experiment is shown on Figure S3.

We decided to increase the size of the tails by fusing them to a known protein, unrelated to the influenza virus life cycle. A larger ligand should allow the experiment to be performed with lower ligand densities, resulting in lower mass transport rates. We chose the yellow fluorescent protein because of its small size and compact folding, with an unchanged capture strategy on the biosensor surface. A YFP construct was fused in N_terminal_ for C/NP_TAIL_ and D/NP_TAIL_ and in C_terminal_ for A/NP_TAIL_ and B/NP_TAIL_, to respect their position in the wild-type proteins (Figure 2b). The recombinant proteins were produced, purified (Figure S2) and used in SEC experiments to test their ability to interact with importin-α7. We confirmed that adding the YFP has no visible effect on the interaction between the NP_TAILs_ and the human import factor in solution. For each tail, a peak corresponding to the complex was observed prior those of each separate samples (Figure 3). On the contrary, when the control YFP was mixed with importin-α7, a double peak corresponding to the sum of each sample injected separately was observed (Figure S3).

**Figure 4:**
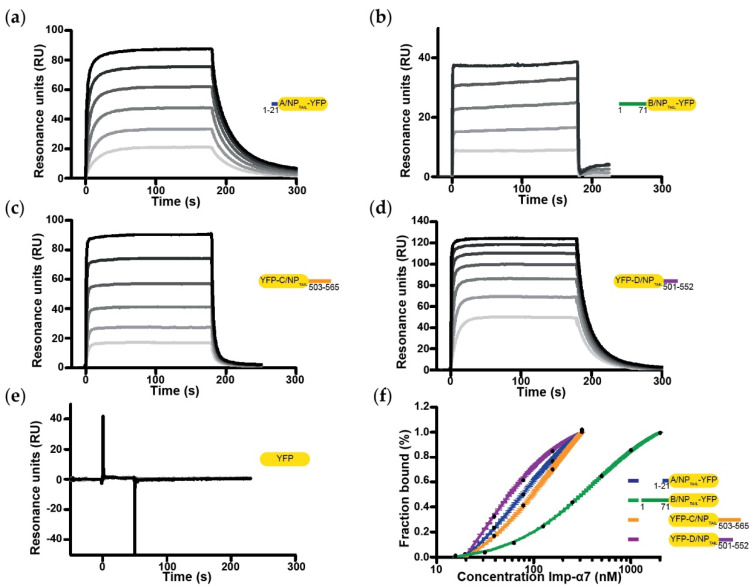
Affinity between YFP-NP_TAILs_ and human importin-α7 by surface plasmon resonance (SPR). The figure shows the sensorgrams of the interaction between importin-α7 and (**a**) A/NP_TAIL_-YFP, (**b**) B/NP_TAIL_-YFP, (**c**) YFP-C/NP_TAIL_, (**d**) YFP-D/NP_TAIL_ and (**e**) the YFP control. For each panel, the gradation of grey represents the different concentrations of importin-α7 used for each titration, from the lowest (light-grey) to the highest (dark-grey). The concentrations range from 19.5 nM to 625 nM for (**a**) and (**c**), from 125 nM to 2 µM for (**b**), from 19.5 nM to 1.25 µM for (**d**) and from 80.5 nM to 10.3 µM for (**e**). (**f**) Steady state analysis was performed on Biacore evaluation software, the resulting fitting normalized and plotted on a log_10_ scale.

Using the corresponding samples, we measured the kinetic parameters of the different interactions by SPR (Figure 4; Table 2 and Table 3 respectively for affinities (*K_d_*) and kinetic parameters values (*k_on_* and *k_off_*)). Gradient concentration of importin-α7 were injected into the SPR flowing channels at a speed of 30/µL·min^−1^ for 50–180/s to reach equilibrium. As shown in Figure 4, a binding signal was generated immediately after injection of importin-α7 which dissociated from the sensor chip very quickly, suggesting that importin-α7 bound to the immobilized NP_TAILs_ in a quick “in and out” manner. Equilibrium analysis was then chosen to derive affinity binding constants (*K_d_*) for the interaction between the immobilized NP_TAILs_ and the importin-α7 in solution. Our reference was the data obtained for the interaction between D/NP_TAIL_ and importin-α7 previously measured using the same method and the same strategy [37] but with a previous generation instrument. Using a Biacore 3000, we had measured a *K_d_* of 99 nM ± 9 nM for this interaction and 29 nM ± 1 nM using a new generation Biacore T200. This time, we were able to fully exploit the SPR data obtained with all NP_TAILs_. In particular, we showed that the architecture of the NLS (mono- or bipartite NLS) has no importance for importin-α7; the affinity for the non-conventional monopartite A/NP_TAIL_ was better than for the bipartite C/NP_TAIL_, with *K_d_* of 73 ± 4 nM and 146 ± 8 nM respectively, but weaker than D/NP_TAIL_. B/NP_TAIL_ appeared to be the lowest value with a *K_d_* of 405 ± 5 nM, but comparable to the value obtained using isothermal titration calorimetry [38]. Despite the nanomolar affinities, all of these interactions displayed a highly dynamic behaviour. Indeed, kinetics parameters show extremely high *on* rates, suggesting that very little energy is needed to form the interaction, but also very high *off* rates. Both association and dissociation rates were close to the upper detection limits of the apparatus, respectively around 10^−6^ M^−1^·s^−1^ and 0.1 s^−1^ and even above in the case of the B/NP_TAIL_ (Table 3).

### 3.3. Systematic Analysis of the Interactions between NP_TAILs_ and Importins-α

Once the strategy for measuring the kinetics of all NP_TAILs_ for the importin-α7 was established, we decided to expand it to other members of the importins-α family. The human genome encodes seven isoforms of importin-α, divided into three subfamilies known as α1, α2 and α3: the α1 subfamily contains importin-α1 and -α8; the α2 subfamily contains importin-α3 and -α4; and the α3 subfamily contains importin-α5, -α6 and -α7 [18,49]. We chose to work with one representative isoform of each subfamily (importin-α1 and importin-α3, respectively for the α1 and α2 subfamilies) plus a second isoform for the α3 subfamily (i.e., importin-α5). Furthermore, all these isoforms are described in the literature regarding the influenza virus infection. We produced and purified the recombinant isoforms without their importin-β binding (IBB) domain. The case of importin-α1 is shown below. Using SEC-MALLS-RI experiments, we first confirmed that both ΔIBB importins-α3 and -α5 are monomeric in solution (Figure S4), before we used them in our SPR experiments to determine their respective kinetics (Table 2 and Table 3). Importin-α5 shows a similar interaction profile as importin-α7, with *K_d_* D/NP_TAIL_ < A/NP_TAIL_ < C/NP_TAIL_ < B/NP_TAIL_ but with a higher amplitude in the values (from 21 to 770 nM). On the contrary, the architecture of the NLS seems essential for recognition by importin-α3. The two bipartite NLS tails (i.e., C/NP_TAIL_ and D/NP_TAIL_) have a higher affinity than the two monopartite (i.e., A/NP_TAIL_ and B/NP_TAIL_) which both show identical values. Based on these results, we further deciphered the interactions by introducing point mutations in the positive charge patches of the NLSs which we had analyzed in the past (i.e., B/NP_TAIL_ and D/NP_TAIL_) [37,38]. We show that for bipartite NLS, the second basic patch could be stronger in relation to the stability of the cargo: importin-α than the first patch (Table 4). Based on the affinities of each single patch mutants, D/NP_TAIL_ seems to present a cooperative behaviour in importin-α binding. On the other hand, importin-α7 seems to be more prompt in compensating for mutations in the first patch whereas importin-α3 looks more permissive to mutations in the second. The results obtained with B/NP_TAIL_ mutant confirm our nuclear magnetic resonance (NMR) observations on the major contribution of the whole tail, from residues 30 to 71, in the interaction with importins-α, and not only the regions surrounding the KR motif [38]. Regarding the B/NP_TAIL mut1_-YFP, even if we were not able to quantify precisely the equilibrium dissociation constants from the raw data, probably because importin-α require a longer injection period to saturate all the ligand binding sites in this condition, the sensorgrams show clear concentration dependent-responses in contrast to the YFP-D/NP_TAIL mut3_ construct (Figure S5).

### 3.4. The Case of the Importin-α1

**Figure 5:**
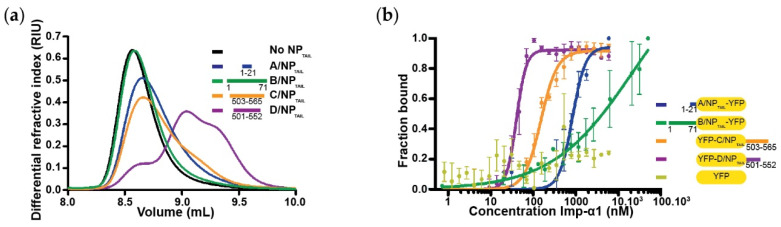
Binding of influenza nucleoproteins tails to importin-α1. (**a**) Dissociation of importin-α1 dimers by competition with NP_TAIL_ using a SEC-MALLS-RI experiments. Human importin-α1 (45 µM) alone or incubated with each NP_TAIL_ (45 µM) was injected on a Superdex^TM^ 75 increase 10/300 G. Successful competition resulted in a delayed elution volume compared to the importin-α1 alone. Three distinct species can be identified from importin-α1:D/NP_TAIL_ injection and two species from importin-α1:C/NP_TAIL_ one. (**b**) YFP-NP_TAILs_: importin-α1 affinities by fluorescence anisotropy. Titration with each NP_TAIL_ (25 nM) was carried out by fluorescence anisotropy with importin-α1 concentrations ranging from 0.7 nM to 50 µM for B/NP_TAIL_-YFP or from 0.8 nM to 6 µM for the other constructs. The titrations were normalized and fitted with GraphPad analysis model “specific binding with Hill slope”. The raw data are shown in Figure S6.

The SEC-MALLS-RI experiments showed a different behaviour of importin-α1 in solution (Figure S4). Whereas the experimental molecular mass for ΔIBB importins-α3, -α5 and _–_α7 were similar to the theoretical values, the results obtained with ΔIBB importin-α1 suggest a propensity for dimerization of this construct. The corresponding sample was eluted precociously from the S200 column with an apparent molecular mass of 88 kDa rather than the theoretical 50 kDa (Figure S4a). Dimerization was reported previously [50] with a monomer–dimer *K_d_* estimated to be 8 μM and the equilibrium could be shifted to the monomeric form by adding NLS-mimicking peptides. In a second SEC-MALLS-RI set of experiments but using a Superdex 75 column, we showed that with the different NP_TAIL_ peptides the peaks corresponding to the importin-α1 were shifted to the smaller molecular mass (Figure 5a). The largest effect is observed with D/NP_TAIL_ suggesting that a quasi-quantitative estimation of the interaction could be extracted from these results, with apparent *K_d_* D/NP_TAIL_ < C/NP_TAIL_ ≤ A/NP_TAIL_ < B/NP_TAIL_.

When we tried to measure the corresponding kinetics by SPR, we could not obtain a stable equilibrium, probably due to the competition between the monomer-dimer transition of importin-α1 and its interaction with the immobilized tails. This experimental artifact is inherent to the nature of the analyte, which despite the purification steps may dimerize in time and significantly affect affinity measurements by SPR. We then found an alternative method; with the YFP-NP_TAILs_ we could measure the fluorescence anisotropy. Fluorescence anisotropy is a measure of light emitted by a fluorophore, unequal along different axes of polarization. This measure depends on rotational behaviour of this fluorescent molecule in solution. Depending on the fluorophore environment, fluorescence anisotropy will vary from a low value because of its high rotational rate when the molecule is free, to a high value in case of its interaction with a partner. This feature allows the determination of an apparent binding constant when the fluorescent molecule is kept at a constant concentration upon titration of its partner. By titration of our ΔIBB importin-α1 construct in presence of the different YFP-NP_TAILs_ (Figure 5b), the corresponding apparent *K_d_* was determined (Table 5). The curve of the B/NP_TAIL_ could not be estimated because the *K_d_* of the B/NP_TAIL_ for importin-α1 was too close to the *K_d_* of the dimerization of importin-α1.

## 4. Discussion

The interaction between viral proteins and cellular factors is of growing interest for understanding the evolution mechanisms of viruses, and RNA viruses in particular. These are major potential therapeutic targets, as these interfaces are less prone to mutations. It has been decades since the interactions between importin-α and the influenza virus nucleoprotein have been studied but numerous features and mechanisms are still unknown [32,33,35,51]. Importins-α are essential actors in cells and were diversified and highly conserved through evolution (Table S2). Each importin-α shows a high sequence identity in higher eukaryotes with a specific expression pattern and behaviour but also preferential bindings to cargo and functions.

In this paper, we provide a comparative characterization of the interactions between four importin-α isoforms with the tails from A/NP, B/NP, C/NP and D/NP. The affinities are between the nanomolar and the low micromolar range, with association and dissociation rates surprisingly high. Two different behaviours were identified: (i) A/NP_TAIL_, C/NP_TAIL_ and D/NP_TAIL_ present a dynamic complex formation and release with high-affinity preferential binding and (ii) B/NP_TAIL_ shows even faster association and dissociation rates with very low affinity.

The A/NP_TAIL_ was isolated from a human strain of influenza A virus and interacted preferentially with importin-α7. This is consistent with the importins-α expression pattern in the human respiratory track [52] as well as of previously reported work [31]. It is of note that PB2 isolated from human strains also preferentially bind to importin-α7, while avian PB2s tend to interact with importin-α3 [28]. The poor binding of A/NP_TAIL_ to importin-α3 is consistent with the fact that importin-α3 tends not to interact well with monopartite NLS; while A/NP_TAIL_ is not a monopartite NLS *per se*, it possesses only a short NLS motif. In contrast, C/NP_TAIL_ presents a higher affinity for importin-α3. Importin-α3 has a low auto-inhibition and a unique intrinsic flexibility, acting as an adapter for unusual and complex NLS sequences or NLS close to globular domains [53,54]. Such NLSs could not associate with a less flexible importin-α due to steric hindrance. The NLS sequence of C/NP_TAIL_ is similar to a bipartite NLS, with two basic patches, but separated by a long linker sequence (more than 20 residues). Such a sequence could require the flexibility of importin-α3 to achieve optimal binding. D/NP_TAIL_ is a textbook case of bipartite NLS, which binds tightly to importins-α, using both the minor and major binding pockets [55,56,57]. D/NP_TAIL_ strongly interacts with all tested importins with a high affinity (20–41 nM), in the same range as other bipartite NLSs (*K_d_* ≈ 10 nM) [28,58]. In other studies, it has been shown that the in vitro affinities using purified proteins and peptides were in the nanomolar range whereas the interaction between NLSs and importins-α *in cellulo* is in the micromolar range. Addition of cytosol in in vitro assays led to the destabilization of importin: NLS complexes, even in the case of bacterial lysate, highlighting the contribution of non-specific interaction and competition in this cellular process [44,59]. Importins cycles between both compartments have been estimated to be fast, around 30 ms; nanomolar affinity leads to a residence time of several seconds or up to several minutes, which is far too long to allow optimal release in the nucleus, while micromolar affinity put it in the millisecond range [59].

The sensorgram shapes of C/NP_TAIL_ and D/NP_TAIL_ along with the affinities resulting from D/NP_TAIL_ mutations clearly suggest that the interaction follows a 1:1 binding model with cooperativity. At the other end of the spectrum, B/NP_TAIL_ behaviour towards importins-α is still puzzling. It is possible that the B/NP_TAIL_ is not properly processed in our settings. It is well known that A/NP and B/NP nuclear import are regulated through post-translational modifications (PTM), in particular through phosphorylation [60,61]. A discrete PTM could be an activating signal for nuclear import regulation [62,63,64,65,66]. The absence of the NP_CORE_ in our experiment could also be responsible for the lack of functionality of B/NP_TAIL_: three-dimensional context could matter for importin specificity, with putative additional contacts and constrains, and for NLS docking [18,28,53,67]. Another hypothesis comes from the nuclear import receptor involved and the complex to be imported. While importin-α/β complex is largely believed to be the most common nuclear import pathway, this is still debated. Other adaptor proteins besides importin-α are known to interact with cargo and importin-β [68,69]. Co-import of neo-synthesized B/NP with another cargo is also a possibility, taking in account that RNP nuclear import functionality putatively relies on avidity rather than affinity [70,71]. Therefore, it is not be excluded that B/NP uses another receptor/complex to gain access to the nucleus. Further investigations into other nuclear import receptors could shed light on successful B/NP nuclear translocation. One interesting feature of B/NP is its long, intrinsically disordered N_terminal_ tail. Such a tail could be involved in the formation of liquid phase separation and help with protein recruitment [72,73]. If B/NP exhibits such behaviour, it could be interesting to dissect it further as nucleoporins in the nuclear pore complex channel tend themselves to be involved in phase separation [74,75,76].

Our work focuses on human importins-α, as three out of the four NP_tails_ were derived from human viruses. However, due to the high conservation of these importins through evolution (Table S2), it is likely that extrapolation to other mammal importins can be achieved to some degree. Further work would be required to validate this completely.

While a lot of work is still required to fully understand the mechanistic and specificity of the interactions between influenza nucleoproteins and importins-α, we provide here this first comparative study on the interaction of NP_TAILs_ with importins-α1, -α3, -α5 and -α7. NP_CORE_ involvement, NP oligomerization, cellular environment, IBB domain contribution, and post-translational modification of both partners are other factors which need to be investigated in the future, in the hope of fully dissecting this host-pathogen interaction and trying to target and disrupt this essential hijacking mechanism.

## 5. Conclusions

While the interactions between influenza NPs and importins-α have been extensively studied, this has mainly been done through the prism of A/NP, with a large number of highly different methods and sometimes without the essential focus required on importins-α. Whereas each method and study brought useful characterization insights, it is a complex task to compare affinity from widely different assays, with significant changes in conditions, partners, settings and analysis. Here, we provide the first comparative study to dissect several interactions between influenza NPs and importins-α using the same strategy and under the same conditions. We showed that A/NP_TAIL_, C/NP_TAIL_ and D/NP_TAIL_ interact strongly with one or several importins-α. On the contrary, the human specific B/NP_TAIL_ interacts poorly with selected importins-α, raising new questions about this essential interaction in the influenza virus cycle.

## Figures and Tables

**Table 1:** Binding of influenza nucleoproteins or their peptides to importins-α according to the literature. Abbreviations: ITC, isothermal titration calorimetry; SPBA, solid phase binding assay; MT, microscale thermophoresis; SPR, surface plasmon resonance.

	Construct	Importin-α	Method	K_d_	Ref
A/NP	R416A	α5	ITC	26 nM	[41]
A/NP	2–15	α1	SPBA	1.7 µM	[42]
A/NP	1–13	α1	ITC	5 µM	[31]
A/NP	198–216	α1	ITC	72 µM	[31]
B/NP	1–70	α7	ITC	844 nM	[38]
C/NP	506–565	α1	MT	48 nM	[43]
D/NP	505–552	α7	SPR	100 nM	[37]

**Table 2:** Affinity between wild-type influenza nucleoprotein tails and human importins-α obtained by SPR. The apparent dissociation constants (*K_d_*) are expressed in nM; n.i. means ‘no interaction’.

	Imp-α3	Imp-α5	Imp-α7
A/NP_TAIL_	301 ± 21	128 ± 20	73 ± 4
B/NP_TAIL_	308 ± 106	770 ± 24	405 ± 5
C/NP_TAIL_	59 ± 9	287 ± 31	146 ± 8
D/NP_TAIL_	22 ± 15	21 ± 5	29 ± 1
YFP control	n.i.	n.i.	n.i.

**Table 3:** Kinetic parameters of the interaction between wild-type influenza nucleoprotein tails and human importins-α obtained by SPR. The association rate constants (*k_on_*) are expressed in M^−1^·s^−1^ and the dissociation rate constants (*k_off_*) values are expressed in s^−1^; n.i. means ‘no interaction’.

	Imp-α3		Imp-α5		Imp-α7	
	*k_on_* (×10^6^)	*k_off_* (×10^−1^)	*k_on_* (×10^6^)	*k_off_* (×10^−1^)	*k_on_* (×10^6^)	*k_off_* (×10^−1^)
A/NP_TAIL_	1.31 ± 0.03	2.81 ± 0.04	1.0 ± 0.6	1.6 ± 0.1	1.6 ± 0.1	0.7 ± 0.1
B/NP_TAIL_	>10	>10	>10	>10	>10	>10
C/NP_TAIL_	2.4 ± 1.1	1.3 ± 0.4	6.1 ± 3.6	2.6 ± 0.4	2.6 ± 0.4	2.5 ± 0.1
D/NP_TAIL_	2.8 ± 0.2	0.07 ± 0.01	1.1 ± 0.1	5.0 ± 0.2	5.0 ± 0.2	1.04 ± 0.03
YFP control	n.i.	n.i.	n.i.	n.i.	n.i.	n.i.

**Table 4:** Effects of mutations in B/NP_TAIL_ and D/NP_TAIL_ on the affinities for the human importins-α. The *K_d_* obtained by SPR are expressed in nM; n.i. means ‘no interaction’.

	Imp-α3	Imp-α5	Imp-α7
B/NP_TAIL_	308 ± 106	770 ± 24	405 ± 5
B/NP_TAIL mut1_	>5000	>5000	>5000
D/NP_TAIL_	22 ± 15	21 ± 5	29 ± 1
D/NP_TAIL mut1_	922 ± 77	1150 ± 154	658 ± 208
D/NP_TAIL mut2_	2457 ± 184	>5000	>5000
D/NP_TAIL mut3_	n.i.	n.i.	n.i.
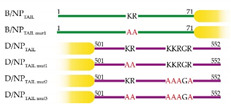			

**Table 5:** Affinity between wild-type influenza nucleoprotein tails and human importin-α1 obtained by fluorescence anisotropy. The apparent *K_d_* are expressed in nM; n.d. and n.i. mean ‘not determined’ and ‘no interaction’, respectively.

	A/NP_TAIL_	B/NP_TAIL_	C/NP_TAIL_	D/NP_TAIL_	YFP Control
imp-αl	621 ± 89	n.d.	149 ± 1	41 ± 12	n.i.

## Supplementary Materials

The following are available online at https://www.mdpi.com/1999-4915/12/8/834/s1, Figure S1: analysis of the complexes between NP_TAILS_ and human importin-α7 in solution, Figure S2: SEC-MALLS-RI analysis of the different YFP-fused tails used in that work, Figure S3: absence of interaction between YFP and human importin-α7 in solution, Figure S4: SEC-MALLS-RI analysis of the different importins-α used in that work, Figure S5: interaction between human importin-α7 and YFP-NP_TAILs_ mutants, Figure S6: interaction between human importin-α1 and YFP-NP_TAILs_, Table S1: sequences, Table S2: sequence identity between the importins-α used in the studies.

## References

[1] Shi M., Lin X.D., Chen X., Tian J.H., Chen L.J., Li K., Wang W., Eden J.S., Shen J.J., Liu L. (2018). The evolutionary history of vertebrate RNA viruses. Nature.

[2] Klumpp K., Ruigrok R.W., Baudin F. (1997). Roles of the Influenza virus polymerase and nucleoprotein in forming a functional RNP structure. EMBO J..

[3] Coloma R., Arranz R., de la Rosa-Trevin J.M., Sorzano C.O.S., Munier S., Carlero D., Naffakh N., Ortin J., Martin-Benito J. (2020). Structural insights into Influenza A virus ribonucleoproteins reveal a processive helical track as transcription mechanism. Nat. Microbiol..

[4] Feldherr C.M., Kallenbach E., Schultz N. (1984). Movement of a karyophilic protein through the nuclear pores of oocytes. J. Cell Biol..

[5] Davis L.I. (1995). The nuclear pore complex. Annu. Rev. Biochem..

[6] Mans B.J., Anantharaman V., Aravind L., Koonin E.V. (2004). Comparative genomics, evolution and origins of the nuclear envelope and nuclear pore complex. Cell Cycle.

[7] Lin D.H., Hoelz A. (2019). The structure of the nuclear pore complex (an update). Annu. Rev. Biochem..

[8] Bonner W.M. (1975). Protein migration into nuclei. II. Frog oocyte nuclei accumulate a class of microinjected oocyte nuclear proteins and exclude a class of microinjected oocyte cytoplasmic proteins. J. Cell Biol..

[9] Chook Y.M., Suel K.E. (2011). Nuclear import by karyopherin-betas: Recognition and inhibition. Biochim. Biophys. Acta.

[10] Timney B.L., Raveh B., Mironska R., Trivedi J.M., Kim S.J., Russel D., Wente S.R., Sali A., Rout M.P. (2016). Simple rules for passive diffusion through the nuclear pore complex. J. Cell Biol..

[11] Gorlich D., Vogel F., Mills A.D., Hartmann E., Laskey R.A. (1995). Distinct functions for the two importin subunits in nuclear protein import. Nature.

[12] Chook Y.M., Blobel G. (2001). Karyopherins and nuclear import. Curr. Opin. Struct. Biol..

[13] Lange A., Mills R.E., Lange C.J., Stewart M., Devine S.E., Corbett A.H. (2007). Classical nuclear localization signals: Definition, function, and interaction with importin alpha. J. Biol. Chem..

[14] Lee B.J., Cansizoglu A.E., Suel K.E., Louis T.H., Zhang Z., Chook Y.M. (2006). Rules for nuclear localization sequence recognition by karyopherin beta 2. Cell.

[15] Bono F., Cook A.G., Grunwald M., Ebert J., Conti E. (2010). Nuclear import mechanism of the EJC component Mago-Y14 revealed by structural studies of importin 13. Mol. Cell.

[16] Padavannil A., Sarkar P., Kim S.J., Cagatay T., Jiou J., Brautigam C.A., Tomchick D.R., Sali A., D’Arcy S., Chook Y.M. (2019). Importin-9 wraps around the H2A-H2B core to act as nuclear importer and histone chaperone. eLife.

[17] Swale C., Da Costa B., Sedano L., Garzoni F., McCarthy A.A., Berger I., Bieniossek C., Ruigrok R.W.H., Delmas B., Crepin T. (2020). X-ray structure of the human karyopherin RanBP5, an essential factor for Influenza polymerase nuclear trafficking. J. Mol. Biol..

[18] Pumroy R.A., Cingolani G. (2015). Diversification of importin-alpha isoforms in cellular trafficking and disease states. Biochem. J..

[19] Oka M., Yoneda Y. (2018). Importin alpha: Functions as a nuclear transport factor and beyond. Proc. Jpn. Acad. Ser. B Phys. Biol. Sci..

[20] Martin K., Helenius A. (1991). Transport of incoming Influenza virus nucleocapsids into the nucleus. J. Virol..

[21] Neumann G., Castrucci M.R., Kawaoka Y. (1997). Nuclear import and export of Influenza virus nucleoprotein. J. Virol..

[22] Deng T., Engelhardt O.G., Thomas B., Akoulitchev A.V., Brownlee G.G., Fodor E. (2006). Role of ran binding protein 5 in nuclear import and assembly of the Influenza virus RNA polymerase complex. J. Virol..

[23] Hutchinson E.C., Orr O.E., Man Liu S., Engelhardt O.G., Fodor E. (2011). Characterization of the interaction between the Influenza A virus polymerase subunit PB1 and the host nuclear import factor Ran-binding protein 5. J. Gen. Virol..

[24] Swale C., Monod A., Tengo L., Labaronne A., Garzoni F., Bourhis J.M., Cusack S., Schoehn G., Berger I., Ruigrok R.W. (2016). Structural characterization of recombinant IAV polymerase reveals a stable complex between viral PA-PB1 heterodimer and host RanBP5. Sci. Rep..

[25] Tarendeau F., Boudet J., Guilligay D., Mas P., Bougault C., Boulo S., Baudin F., Ruigrok R.W.H., Daigle N., Ellenberg J. (2007). Structure and nuclear import function of the C-terminal domain of Influenza virus polymerase PB2 subunit. Nat. Struct. Mol. Biol..

[26] Boivin S., Hart D.J. (2011). Interaction of the Influenza A virus polymerase PB2 C-terminal region with importin alpha isoforms provides insights into host adaptation and polymerase assembly. J. Biol. Chem..

[27] Hudjetz B., Gabriel G. (2012). Human-like PB2 627K Influenza virus polymerase activity is regulated by importin-alpha1 and -alpha7. PLoS Pathog..

[28] Pumroy R.A., Ke S., Hart D.J., Zachariae U., Cingolani G. (2015). Molecular determinants for nuclear import of Influenza A PB2 by importin alpha isoforms 3 and 7. Structure.

[29] Cros J.F., García-Sastre A., Palese P. (2005). An unconventional NLS is critical for the nuclear import of the Influenza A virus nucleoprotein and ribonucleoprotein. Traffic.

[30] Weber F., Kochs G., Gruber S., Haller O. (1998). A classical bipartite nuclear localization signal on Thogoto and Influenza A virus nucleoproteins. Virology.

[31] Wu W., Sankhala R.S., Florio T.J., Zhou L., Nguyen N.L.T., Lokareddy R.K., Cingolani G., Pante N. (2017). Synergy of two low-affinity NLSs determines the high avidity of Influenza A virus nucleoprotein NP for human importin alpha isoforms. Sci. Rep..

[32] O’Neill R.E., Palese P. (1995). NPI-1, the human homolog of SRP-1, interacts with Influenza virus nucleoprotein. Virology.

[33] Wang P., Palese P., O’Neill R.E. (1997). The NPI-1/NPI-3 (karyopherin alpha) binding site on the Influenza a virus nucleoprotein NP is a nonconventional nuclear localization signal. J. Virol..

[34] Melen K., Fagerlund R., Franke J., Kohler M., Kinnunen L., Julkunen I. (2003). Importin alpha nuclear localization signal binding sites for STAT1, STAT2, and Influenza A virus nucleoprotein. J. Biol. Chem..

[35] Gabriel G., Klingel K., Otte A., Thiele S., Hudjetz B., Arman-Kalcek G., Sauter M., Shmidt T., Rother F., Baumgarte S. (2011). Differential use of importin-alpha isoforms governs cell tropism and host adaptation of Influenza virus. Nat. Commun..

[36] Sasaki Y., Hagiwara K., Kakisaka M., Yamada K., Murakami T., Aida Y. (2013). Importin alpha3/Qip1 is involved in multiplication of mutant Influenza virus with alanine mutation at amino acid 9 independently of nuclear transport function. PLoS ONE.

[37] Donchet A., Oliva J., Labaronne A., Tengo L., Miloudi M., Gerard F.C.A., Mas C., Schoehn G., Ruigrok R.W.H., Ducatez M. (2019). The structure of the nucleoprotein of Influenza D shows that all *Orthomyxoviridae* nucleoproteins have a similar NP_CORE_, with or without a NP_TAIL_ for nuclear transport. Sci. Rep..

[38] Labaronne A., Milles S., Donchet A., Jensen M.R., Blackledge M., Bourhis J.M., Ruigrok R.W.H., Crepin T. (2017). Structural analysis of the complex between Influenza B nucleoprotein and human importin-alpha. Sci. Rep..

[39] Ozawa M., Fujii K., Muramoto Y., Yamada S., Yamayoshi S., Takada A., Goto H., Horimoto T., Kawaoka Y. (2007). Contributions of two nuclear localization signals of Influenza A virus nucleoprotein to viral replication. J. Virol..

[40] Ng A.K., Zhang H., Tan K., Li Z., Liu J.H., Chan P.K., Li S.M., Chan W.Y., Au S.W., Joachimiak A. (2008). Structure of the Influenza virus A H5N1 nucleoprotein: Implications for RNA binding, oligomerization, and vaccine design. FASEB J..

[41] Boulo S., Akarsu H., Lotteau V., Muller C.W., Ruigrok R.W., Baudin F. (2011). Human importin alpha and RNA do not compete for binding to Influenza A virus nucleoprotein. Virology.

[42] Nakada R., Hirano H., Matsuura Y. (2015). Structure of importin-alpha bound to a non-classical nuclear localization signal of the Influenza A virus nucleoprotein. Sci. Rep..

[43] Tang Y.S., Lo C.Y., Mok C.K., Chan P.K., Shaw P.C. (2019). The extended C-terminal region of Influenza C virus nucleoprotein is important for nuclear import and ribonucleoprotein activity. J. Virol..

[44] Timney B.L., Tetenbaum-Novatt J., Agate D.S., Williams R., Zhang W., Chait B.T., Rout M.P. (2006). Simple kinetic relationships and nonspecific competition govern nuclear import rates *in vivo*. J. Cell Biol..

[45] Mackmull M.T., Klaus B., Heinze I., Chokkalingam M., Beyer A., Russell R.B., Ori A., Beck M. (2017). Landscape of nuclear transport receptor cargo specificity. Mol. Syst. Biol..

[46] Sumarheni S., Hong S.S., Josserand V., Coll J.L., Boulanger P., Schoehn G., Fender P. (2014). Human full-length coagulation factor X and a GLA domain-derived 40-mer polypeptide bind to different regions of the adenovirus serotype 5 hexon capsomer. Hum. Gene Ther..

[47] Wyatt P.J. (1998). Submicrometer particle sizing by multiangle light scattering following fractionation. J. Colloid Interface Sci..

[48] Labaronne A., Swale C., Monod A., Schoehn G., Crepin T., Ruigrok R.W. (2016). Binding of RNA by the nucleoproteins of Influenza viruses A and B. Viruses.

[49] Mason D.A., Stage D.E., Goldfarb D.S. (2009). Evolution of the metazoan-specific importin alpha gene family. J. Mol. Evol..

[50] Miyatake H., Sanjoh A., Unzai S., Matsuda G., Tatsumi Y., Miyamoto Y., Dohmae N., Aida Y. (2015). Crystal structure of human importin-alpha1 (Rch1), revealing a potential autoinhibition mode involving homodimerization. PLoS ONE.

[51] Resa-Infante P., Paterson D., Bonet J., Otte A., Oliva B., Fodor E., Gabriel G. (2015). Targeting importin-alpha7 as a therapeutic approach against pandemic Influenza viruses. J. Virol..

[52] Ninpan K., Suptawiwat O., Boonarkart C., Phuangphung P., Sathirareuangchai S., Uiprasertkul M., Auewarakul P. (2016). Expression of importin-alpha isoforms in human nasal mucosa: Implication for adaptation of avian Influenza A viruses to human host. Virol. J..

[53] Sankhala R.S., Lokareddy R.K., Begum S., Pumroy R.A., Gillilan R.E., Cingolani G. (2017). Three-dimensional context rather than NLS amino acid sequence determines importin alpha subtype specificity for RCC1. Nat. Commun..

[54] Smith K.M., Tsimbalyuk S., Edwards M.R., Cross E.M., Batra J., Soares da Costa T.P., Aragao D., Basler C.F., Forwood J.K. (2018). Structural basis for importin alpha 3 specificity of W proteins in Hendra and Nipah viruses. Nat. Commun..

[55] Robbins J., Dilworth S.M., Laskey R.A., Dingwall C. (1991). Two interdependent basic domains in nucleoplasmin nuclear targeting sequence: Identification of a class of bipartite nuclear targeting sequence. Cell.

[56] Fontes M.R., Teh T., Kobe B. (2000). Structural basis of recognition of monopartite and bipartite nuclear localization sequences by mammalian importin-alpha. J. Mol. Biol..

[57] Hodel M.R., Corbett A.H., Hodel A.E. (2001). Dissection of a nuclear localization signal. J. Biol. Chem..

[58] Lokareddy R.K., Hapsari R.A., van Rheenen M., Pumroy R.A., Bhardwaj A., Steen A., Veenhoff L.M., Cingolani G. (2015). Distinctive properties of the nuclear localization signals of inner nuclear membrane proteins Heh1 and Heh2. Structure.

[59] Cardarelli F., Bizzarri R., Serresi M., Albertazzi L., Beltram F. (2009). Probing nuclear localization signal-importin alpha binding equilibria in living cells. J. Biol. Chem..

[60] Hutchinson E.C., Denham E.M., Thomas B., Trudgian D.C., Hester S.S., Ridlova G., York A., Turrell L., Fodor E. (2012). Mapping the phosphoproteome of Influenza a and B viruses by mass spectrometry. PLoS Pathog..

[61] Zheng W., Li J., Wang S., Cao S., Jiang J., Chen C., Ding C., Qin C., Ye X., Gao G.F. (2015). Phosphorylation controls the nuclear-cytoplasmic shuttling of Influenza A virus nucleoprotein. J. Virol..

[62] Terry L.J., Shows E.B., Wente S.R. (2007). Crossing the nuclear envelope: Hierarchical regulation of nucleocytoplasmic transport. Science.

[63] Bedford M.T., Clarke S.G. (2009). Protein arginine methylation in mammals: Who, what, and why. Mol. Cell.

[64] Nardozzi J., Wenta N., Yasuhara N., Vinkemeier U., Cingolani G. (2010). Molecular basis for the recognition of phosphorylated STAT1 by importin alpha5. J. Mol. Biol..

[65] Wang Y.E., Pernet O., Lee B. (2012). Regulation of the nucleocytoplasmic trafficking of viral and cellular proteins by ubiquitin and small ubiquitin-related modifiers. Biol. Cell..

[66] Ptak C., Wozniak R.W. (2017). SUMO and nucleocytoplasmic transport. Adv. Exp. Med. Biol..

[67] Friedrich B., Quensel C., Sommer T., Hartmann E., Kohler M. (2006). Nuclear localization signal and protein context both mediate importin alpha specificity of nuclear import substrates. Mol. Cell. Biol..

[68] Jullien D., Gorlich D., Laemmli U.K., Adachi Y. (1999). Nuclear import of RPA in *Xenopus* egg extracts requires a novel protein XRIPalpha but not importin alpha. EMBO J..

[69] Narayanan U., Ospina J.K., Frey M.R., Hebert M.D., Matera A.G. (2002). SMN, the spinal muscular atrophy protein, forms a pre-import snRNP complex with snurportin1 and importin beta. Hum. Mol. Genet..

[70] Hodges J.L., Leslie J.H., Mosammaparast N., Guo Y., Shabanowitz J., Hunt D.F., Pemberton L.F. (2005). Nuclear import of TFIIB is mediated by Kap114p, a karyopherin with multiple cargo-binding domains. Mol. Biol. Cell.

[71] Tome-Amat J., Ramos I., Amanor F., Fernandez-Sesma A., Ashour J. (2019). Influenza A virus utilizes low-affinity, high-avidity interactions with the nuclear import machinery to ensure infection and immune evasion. J. Virol..

[72] Schuster B.S., Reed E.H., Parthasarathy R., Jahnke C.N., Caldwell R.M., Bermudez J.G., Ramage H., Good M.C., Hammer D.A. (2018). Controllable protein phase separation and modular recruitment to form responsive membraneless organelles. Nat. Commun..

[73] Guseva S., Milles S., Jensen M.R., Salvi N., Kleman J.P., Maurin D., Ruigrok R.W.H., Blackledge M. (2020). Measles virus nucleo- and phosphoproteins form liquid-like phase-separated compartments that promote nucleocapsid assembly. Sci. Adv..

[74] Milles S., Mercadante D., Aramburu I.V., Jensen M.R., Banterle N., Koehler C., Tyagi S., Clarke J., Shammas S.L., Blackledge M. (2015). Plasticity of an ultrafast interaction between nucleoporins and nuclear transport receptors. Cell.

[75] Zilman A. (2018). Aggregation, phase separation and spatial morphologies of the assemblies of FG nucleoporins. J. Mol. Biol..

[76] Dormann D. (2020). FG-nucleoporins caught in the act of liquid-liquid phase separation. J. Cell Biol..

