# Supplementary material for: Differential Behaviours and Preferential Bindings of Influenza Nucleoproteins on Importins-α
Source: Viruses. 2020 Jul 30;12(8):834. doi: 10.3390/v12080834 (PMC7472415; doi:10.3390/v12080834)
Supplement: Supplementary file 1 [file viruses-12-00834-s001.pdf]

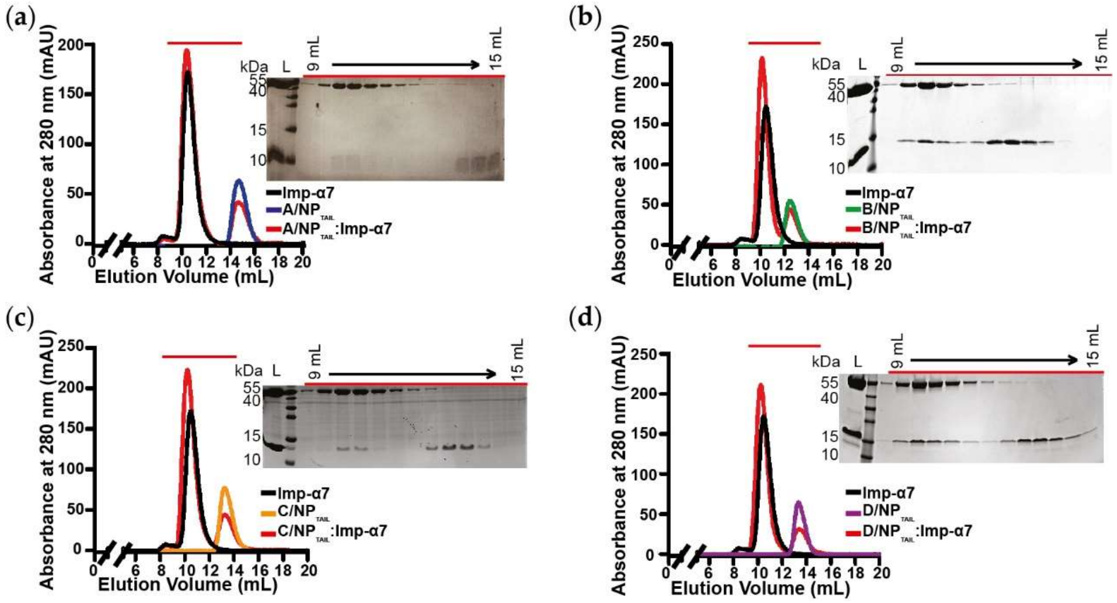

Figure S1. Analysis of the complexes between NPTAILS and human importin-α7 in solution. Each NPTAIL (60 μM) was injected on a Superdex™ 75 10/300GL column, alone or in presence of importin-α7 (25 μM). The figure shows the superimposition of the size exclusion chromatography profiles obtained for (**a**) A/NPTAIL, (**b**) B/NPTAIL, (**c**) C/NPTAIL and (**d**) D/NPTAIL. The colors correspond to the code used on Figure 1, with the complexes in red and the importin-α7 alone in black. For each panel, the 18 % SDS-PAGE corresponding to the elution of the complex is shown.

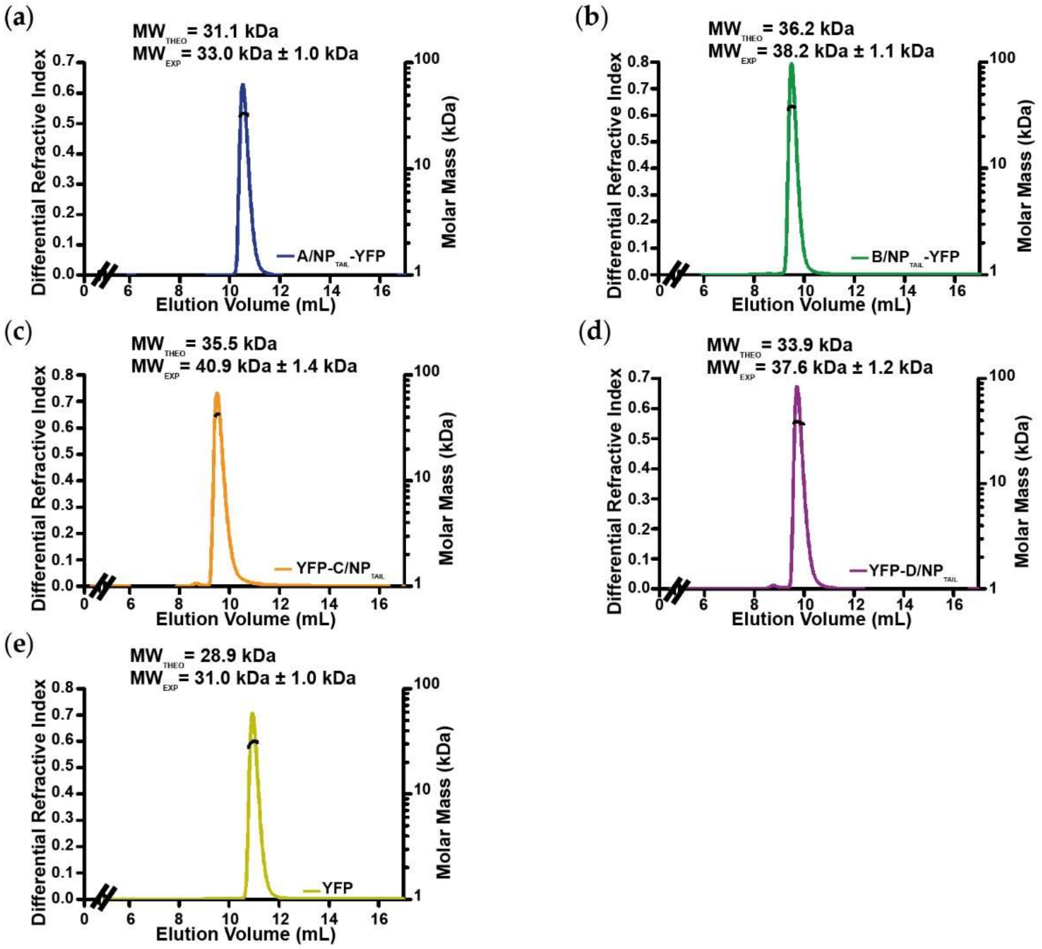

Figure S2. SEC-MALLS-RI analysis of the different YFP-fused tails used in that work. All the samples were injected on a Superdex™ 75 10/300GL column. Each panel indicates the theoretical (top) and experimental (bottom) molecular weights of (**a**) A/NPTAIL-YFP, (**b**) B/NPTAIL-YFP, (**c**) YFP-C/NPTAIL, (**d**) YFP-D/NPTAIL and (**e**) the control YFP. The black lines indicate the molecular weight as estimated below the peak.

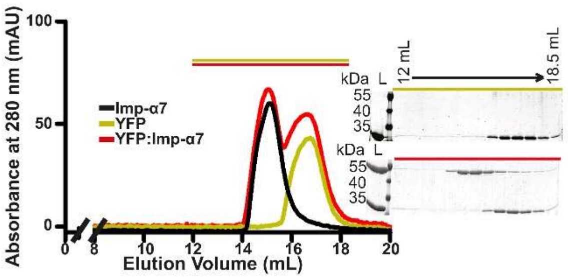

Figure S3. Absence of interaction between YFP and human importin-α7 in solution. The YFP (30 μM) was injected on a Superdex™ 200 increase 10/300GL column, alone or in presence of importin-α7 (25 μM). The 12 % SDS-PAGE corresponding to the elution of the YFP alone (top) and the sample containing both YFP and importin-α7 (bottom) are shown.

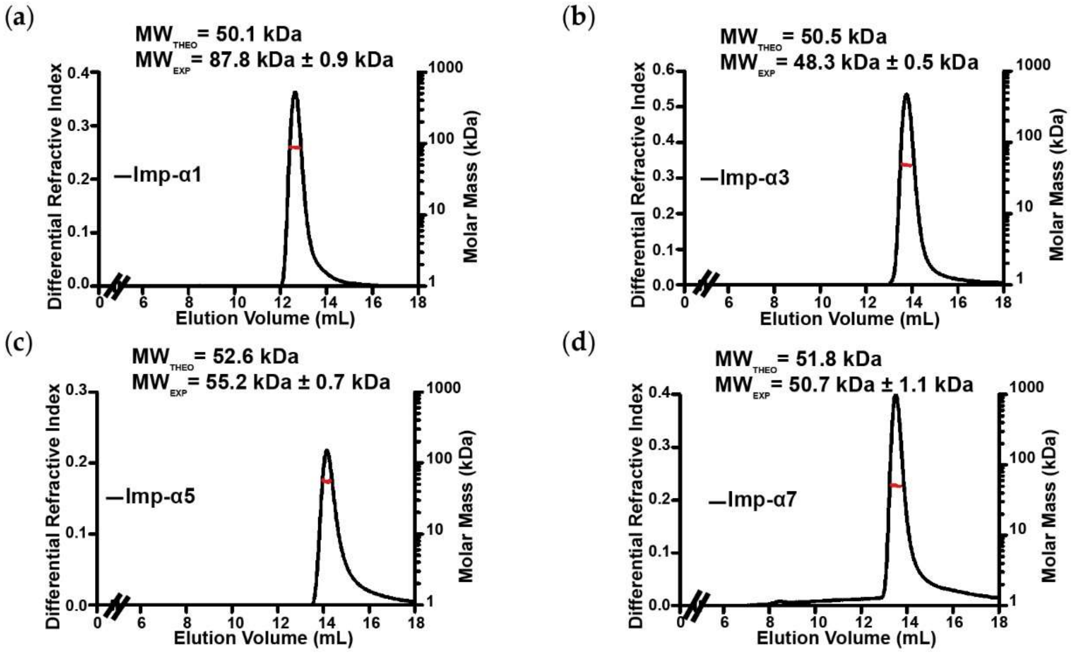

Figure S4. SEC-MALLS-RI analysis of the different importins-α used in that work. All the samples were injected on a Superdex™ 200 increase 10/300GL column. Each panel indicates the theoretical (top) and experimental (bottom) molecular weights of (**a**) importin-α1, (**b**) importin-α3, (**c**) importin-α5 and (**d**) importin-α7. The red lines indicate the molecular weight as estimated below the peak.

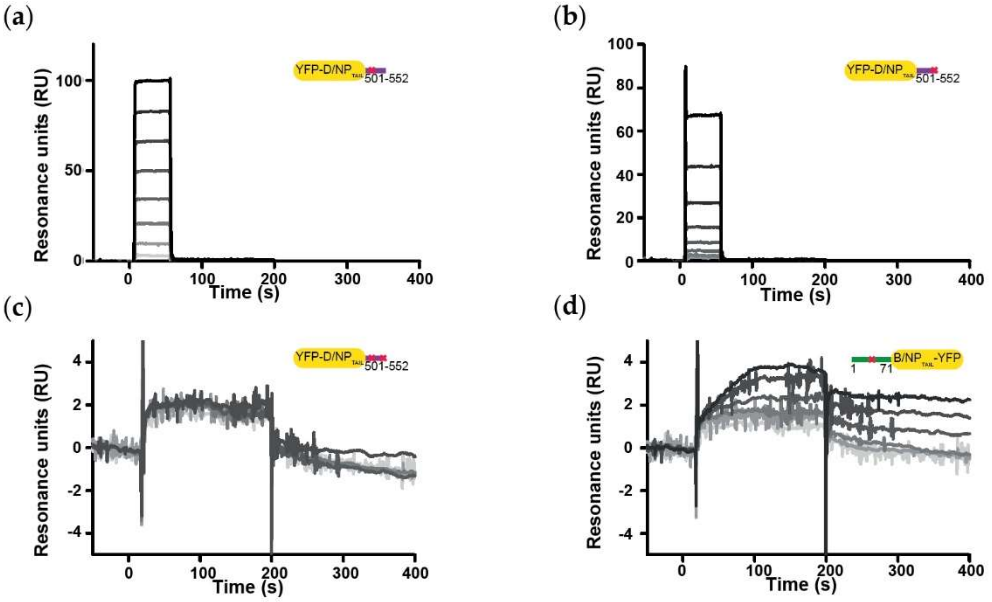

Figure S5. Interaction between human importin-α7 and YFP-NP$_{\text{TAILs}}$ mutants. The figure shows the sensorgrams of the interaction between importin-α7 and (a) YFP-D/NP$_{\text{TAIL mut1}}$, (b) YFP-D/NP$_{\text{TAIL mut2}}$, (c) YFP-D/NP$_{\text{TAIL mut3}}$, and (d) YFP-B/NP$_{\text{TAIL mut1}}$. For each panel, the gradation of grey represents the different concentrations of importin-α7 used for the titrations, from the lowest (light-grey) to the highest (dark-grey). The concentrations range from 80 nM to 10.3 µM for (a) and (c) and from 41 nM to 4 µM for (c) and (d). For a more accurate comparison, (c) and (d) sensorgrams were recovered from the same kinetic assay.

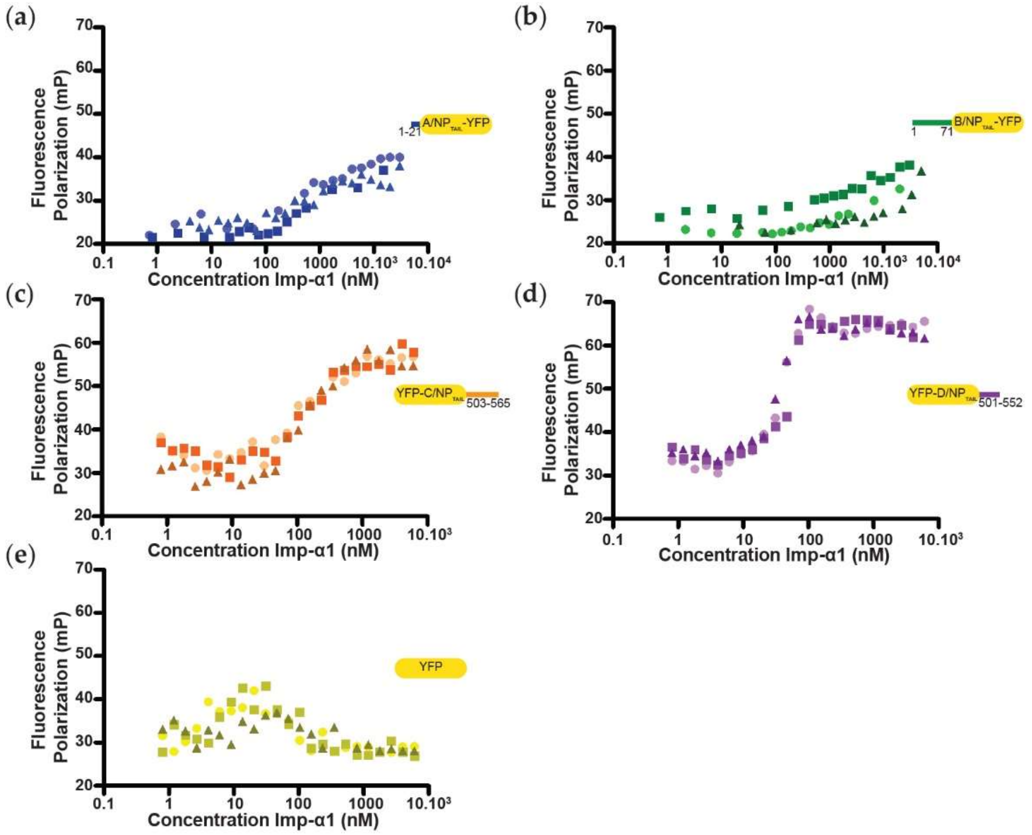

Figure S6. Interaction between human importin-α1 and YFP-NPTAILs. The figure shows the fluorescence polarization raw data of the interaction between importin-α1 and (**a**) A/NPTAIL-YFP, (**b**) B/NPTAIL-YFP, (**c**) YFP-C/NPTAIL, (**d**) YFP-D/NPTAIL and (**e**) YFP control. For each panel, one shade of colour with one specific symbol represent one of the three replicate.

Table S1: Sequences. The table details the sequences of the nucleoproteins tails and biotinylation motif used in this work. The basic residues of the tails are shown in blue and the corresponding mutations in red. Biotinylated lysine are in green.

**A/NP$_{TAIL}$**	
WT	$_{1}$MATKGTKRSYEQMETDGERQN$_{21}$
**B/NP$_{TAIL}$**	
WT	$_{1}$MSNMDIDGINTGTIDKAPEEITSGTSGTTRPIIRPATLAPPSNKRTRNPSPERATTISEADVGRKTQKKQT$_{71}$
B/NP$_{TAIL\ mut1}$	$_{1}$MSNMDIDGINTGTIDKAPEEITSGTSGTTRPIIRPATLAPPSNAATRNPSPERATTISEADVGRKTQKKQT$_{71}$
**C/NP$_{TAIL}$**	
WT	$_{503}$FEFDPDYNPIRVKRPKKPIAKRNSNISRLEEEGMDENSEIGQAKKMKPLDQLTSTSSNIPGKN$_{565}$
**D/NP$_{TAIL}$**	
WT	$_{501}$FEFTGSDVPRTGAKRRVGGADDVTLGTSQPKKRGRQGAGVESSMDIETVGED$_{552}$
D/NP$_{TAIL\ mut1}$	$_{501}$FEFTGSDVPRTGAAARVGGADDVTLGTSQPKKRGRQGAGVESSMDIETVGED$_{552}$
D/NP$_{TAIL\ mut2}$	$_{501}$FEFTGSDVPRTGAKRRVGGADDVTLGTSQPAAAGAQGAGVESSMDIETVGED$_{552}$
D/NP$_{TAIL\ mut3}$	$_{501}$FEFTGSDVPRTGAAARVGGADDVTLGTSQPAAAGAQGAGVESSMDIETVGED$_{552}$
**Synthetic peptide**	
control	$_{1}$LEEMKKGHLERECMEETCSYEEAREVFEDSEKTNEFWNK$_{39}$
**Biotinylation sequences**	
N-terminal	DIFEAQKIEWHEGGNGSGGGLN
C-terminal	NGSGGGLNDIFEAQKIEWHE

Table S2: Sequence identity between the importins-α used in the studies. Sequences and accession numbers were recovered from NCBI/Uniprot databases for human (Homo sapiens) importin-α1 (NP_001307540 / P52292), importin-α3 (NP_002259.1 / O00629), importin-α5 (NP_002255.3 / P52294) and importin-α7 (NP_036448.1 / O60684). Each BLAST was done separately, using one human importin sequence against reference protein sequences from *Sus scrofa* (swine), *Bos Taurus* (bovine), *Gallus gallus* (chicken) and *Anas platyrhynchos* (duck). As no duck importin-α7 was identified in the NCBI database, the corresponding sequence was recovered from Uniprot database (Uniprot accession number U3IY73) and aligned with each human importin alpha to get a sequence identity by global alignment (bold). 99+ corresponds to a sequence identity between 99.5 and 100 %. Importins-α from the same subfamily are highlightened in the same colour.

	Subfamily<br>Member	Human<br>sub-α1<br>Imp-α1	Human<br>sub-α2<br>Imp-α3	Human<br>sub-α3<br>Imp-α5	Human<br>sub-α3<br>Imp-α7
Human	Imp-α1	100	51	46	48
	Imp-α3		100	47	49
	Imp-α5			100	82
	Imp-α7				100
Swine	Imp-α1	97	50	45	47
	Imp-α3	52	99+	47	48
	Imp-α5	46	46	99	81
	Imp-α7	48	48	81	99
Bovine	Imp-α1	95	82	46	47
	Imp-α3	51	99+	47	48
	Imp-α5	46	46	99	81
	Imp-α7	48	48	81	99
Chicken	Imp-α1	83	51	46	47
	Imp-α3	51	99	47	48
	Imp-α5	46	46	96	81
	Imp-α7	48	48	81	94
Duck	Imp-α1	84	51	46	46
	Imp-α3	51	98	47	48
	Imp-α5	46	46	96	81
	Imp-α7	**46**	**48**	**82**	**94**
